# Costs and trade-offs of grazer-induced defenses in *Scenedesmus* under deficient resource

**DOI:** 10.1038/srep22594

**Published:** 2016-03-02

**Authors:** Xuexia Zhu, Jun Wang, Qinwen Chen, Ge Chen, Yuan Huang, Zhou Yang

**Affiliations:** 1 Jiangsu Key Laboratory for Biodiversity and Biotechnology, School of Biological Sciences, Nanjing Normal University, 1 Wenyuan Road, Nanjing 210023, China

## Abstract

The green alga *Scenedesmus obliquus* can form inducible defensive morphs under grazing threat. Costs and trade-offs of inducible defense are expected to accompany the benefits of defensive morphs, but are hard to detect under nutrient-sufficient experimental conditions. To test the existence of costs associated with inducible defense, we cultured *S. obliquus* along resource availability gradients in the presence or absence of infochemical cues from *Daphnia*, and measured the strength of defensive colony formation and fitness characters. Under the lowest phosphorous concentration, the expression of inducible defensive colony resulted in decreased growth rate, which provides direct evidence for physiological costs. Along the gradient reduction of phosphorous concentration or light intensity, inducible defense in *S. obliquus* showed a decreasing trend. However, the photosynthetic efficiency of *S. obliquus* was barely affected by its defense responses, suggesting that the negative correlations between resource availability and colony formation of this alga may be due to resource-based trade-offs in the allocation of limited resources. Thus, our results indicated that expression of inducible defense of *S. obliquus* was impaired under insufficient phosphorus or light. Furthermore, under severe phosphate deficiency, obvious physiological costs of inducible defense could be detected even though defensive colony formation also decreased significantly.

Phytoplankters, which supply a major part of primary productivity in waters, play an important role in aquatic ecosystems. However, several processes, e.g., hydromechanical dispersion, sedimentation, and consumption by grazers, may cause major loss of phytoplankton biomass. Phytoplankters have to avoid sinking out of euphotic zones to maintain access to sufficient light. Therefore, large size may be resisted among phytoplankton to reduce settling velocity^1^. In addition, small algal cells have larger surface-to-volume ratio and shorter internal transport distance. Thus, maintaining a relatively small size will be favored by algae to assimilate inorganic nutrients from the aquatic environment^2^.

Since phytoplankters share an apparently refugeless openwater habitat with zooplankton and phytoplanktivorous fish, consumption by herbivores is another major factor contributing to phytoplankton loss. To avoid being grazed, many taxa of algae in euphotic water have evolved various chemical or physical defensive strategies, such as toxin production, spine or colony formation, and silica plates^3^,^4^,^5^, which allow them to survive in the same area with grazers. Algae’s resistance to herbivores can be either constitutive or inducible. Inducible defenses allow phytoplankton to resist the risk of being grazed under the threat of predation and save extra energy to maintain the defense systems in the absence of predators. The genus *Scenedesmus*, which forms defensive colonies under predation pressure, is one of the genera that can form induced defensive phenotypes in freshwater^5^,^6^. *Scenedesmus* commonly reproduces asexually through the formation of autospores, in which the mother cell divides into 2–16 daughter cells^7^. Daughter cells subsequently form a new overlapping or staggered colony and then break through the parental cell wall, or fail to join and then leave the parental cell wall separately^8^,^9^. The infochemicals released by zooplanktons such as *Daphnia* and *Brachionus* during active grazing processes may trigger the formation of colonies instead of unicells to resist mortality from grazers^6^,^10^. The formation of colony as an anti-herbivore strategy relying on grazing infochemicals is familiar in aquatic ecosystems. Besides *Scenedesmus*, many species can respond to infochemicals released by herbivores feeding on focal algal species, e.g. *Microcystis*^11^, *Coelastrum*^12^. A bloom species *Phaeocystis*, which is abundant in the ocean, can form colonies in response to non-species-specific grazing chemical cues^4^,^13^.

In terms of energy requirements, defenses that are induced only in the presence of herbivores are considered more efficient than constitutive defenses under variable grazing risk^14^. The adaptive benefits of colony formation for *Scenedesmus* spp. have been investigated in many studies^15^,^16^. However, no direct evidence about the cost accompanying with the benefit is detected. Trade-offs and costs associated with grazer-induced colony formation exist theoretically; otherwise, this defensive phenotype would be fixed^17^. Since the energy storage of organisms is limited, excessive investment of energy for defense against grazers would impose restriction on growth and maintenance. For *Scenedesmus*, two main potential indirect costs coupled with morphological defense are identified: reduction of surface-to-volume ratios and enhancement of sinking risk^18^. Both costs would result in loss of competitive advantage in phytoplankton populations. As four, eight, or even more algal cells join together to form a larger resistant coenobium, volume is increased compared to single algal cells, but a high surface-to-volume ratio is sacrificed. In addition, overlapping or staggered coenobiums may result in “package effect” on inner cells, and light capture and nutrient assimilation may be negatively influenced^19^.

Apart from the relatively small surface area for nutrient and light absorption, increased sedimentation rate of large defensive coenobiums also have negative effect on the population of *Scenedesmus*. Colonies not only settle faster but are also less able to resuspend to upper water compared to unicells. For non-motile phytoplankters, prolonged residence in warm upper water with sufficient illumination is a primary requirement. Higher risk of sinking out of euphotic zone increases the loss of population biomass, and decreases the growth rate for light limitation^18^,^20^. Aside from grazing, sedimentation loss appears to be a major threat to small phytoplankton in the pelagic zone^21^. Thus, to preserve high biomass, a trade-off in production of stable large colony may be required to avoid the selective pressure of predation and division into single cells to maximize nutrient uptake in *Scenedesmus*.

Recent research has tried to reveal the cost associated with induced colony formation in *Scenedesmus*^18^,^22^,^23^. However, the direct fitness costs of forming defensive colonies in terms of growth or other physiological indexes^18^,^24^ has been difficult to determine, which may be due to the abundance of nutrients in culture medium used in those studies. Because of the package effect^19^, colonies should have less chlorophyll-specific absorption coefficients than unicells, which should be reflected in the growth rate. However, no significant decrease is observed in growth rate^18^. Forming defensive morphs tends to improve the adaptive fitness of *Scenedesmus*. Nevertheless, when alga is cultured in poor conditions, such as nutritional deficiency or light insufficiency, such conditions may be disadvantageous if the cost of maintaining colonies is high. Colony formation of *S. acutus* has been reported to be a constitutive defense response under phosphorus-sufficient conditions, but an inducible defense response under P-limited conditions^22^, indicating the existence of trade-off between colony formation and nutrient availability. As previous studies have showed, maintaining defensive morphs impairs the competitive ability of *Scenedesmus* under nutrient-limited conditions caused by competition^23^,^25^. Therefore, we hypothesized that when resource is insufficient, the cost of induced defense may be obvious, and a trade-off between defense and resource absorption may emerge. To examine our hypothesis, we measured the induced colony formation, population growth, and photosynthesis of *Scenedesmus* under different phosphorus concentrations and light intensities.

## Results

### Inducible defense of *Scenedesmus* under different phosphorus concentrations

**Figure 1:**
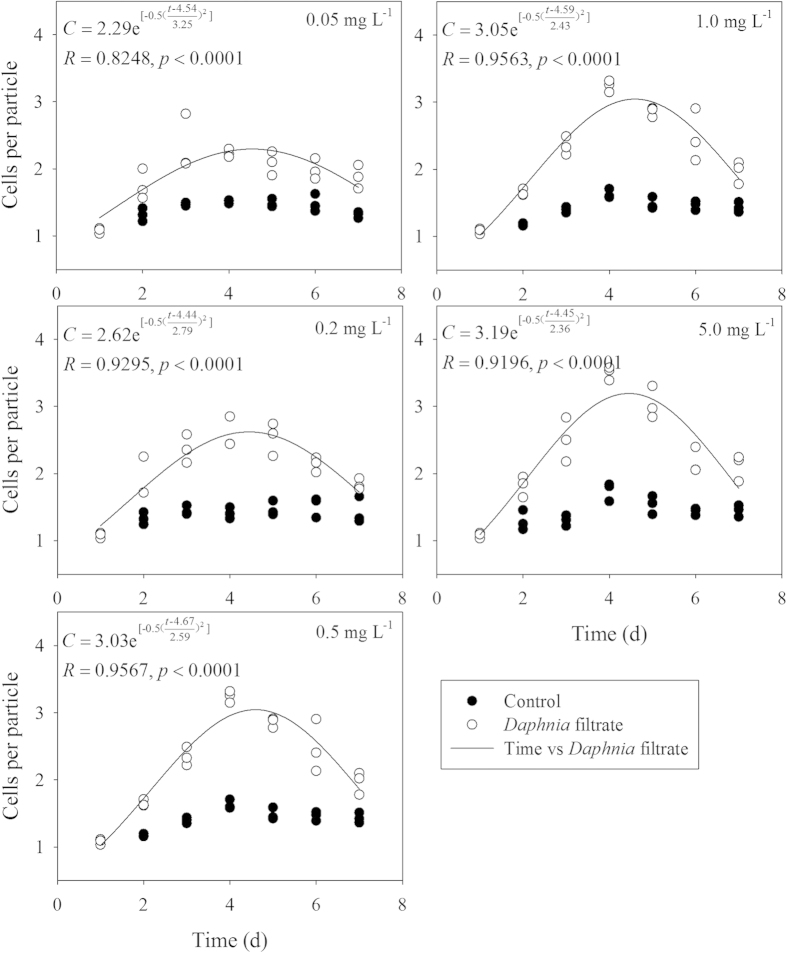
Mean number of cells per particle (*C*) of *Scenedesmus obliquus* in the control or *Daphnia* filtrate groups at different P concentrations. *C* changed with cultural time (*t*) in *Daphnia* filtrate groups fitted by Gaussian distribution.

**Figure 2:**
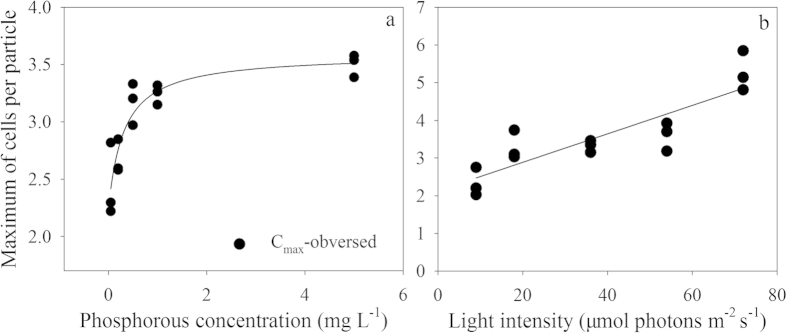
Observed maximum number of cells per particle (*C*) of *S. obliquus* in *Daphnia* filtrate groups at different P concentrations (**a**) and light intensities (**b**).

Addition of *Daphnia* filtrate stimulated the increase of colony size under all five P concentrations (Fig. 1). In the absence of *Daphnia* filtrate, no difference was detected in cells per particle among different P concentrations (F = 1.172, *p* = 0.331). A three-way ANOVA indicated that the induced colony formation was significantly decreased by decrease in P concentration (Table 1). Under the exposure of *Daphnia* infochemicals, the colony size of *S. obliquus* increased dramatically and peaked on day 4 (Fig. 1). Fitted curves revealed that the value of *C*_*max*_ declined, which was caused by decreased P concentration. To investigate the relationship of colony size with resource availability, the observed *C*_*max*_ from each triplicate of the *Daphnia* filtrate treatments was fitted against P concentrations or light intensities (Fig. 2). The dots of *C*_*max*_-observed were the actual maximums. The *C*_*max*_-observed in response to P concentration followed a rectangular hyperbolic model and increased with increasing P concentration, but it no longer increased when the P concentration was above 0.5 mg L^−1^ (Fig. 2a).

**Figure 3:**
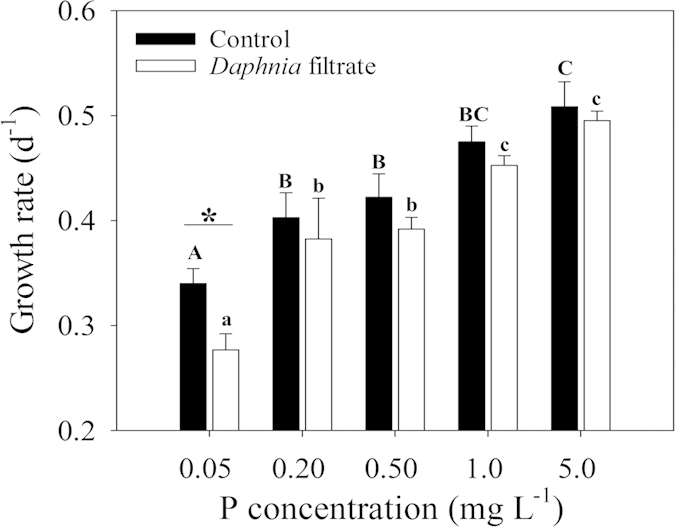
Growth rates of *S. obliquus* in the control and *Daphnia* filtrate groups at different P concentrations. Vertical lines represent ±1 SE (*n* = 3). Different capital letters above solid bars denote significant (*P < 0.05*) differences among the control groups, while different lowercase letters above hollow bars denote significant (*P < 0.05*) differences among *Daphnia* filtrate groups. The asterisk above a short horizontal line indicates significant difference (*P *< 0.05) between control and *Daphnia* filtrate groups under certain P concentrations.

The growth capacity of *S. obliquus* declined with decreased P concentration (Fig. 3). Addition of *Daphnia* filtrate led to significant decline in growth rate in the lowest P concentration (F = 9.188, *p* = 0.039). In other P concentrations, no statistically significant decrease was observed between control and *Daphnia* filtrate groups. A two-way ANOVA was conducted to test the effect of *Daphnia* filtrate and P concentration on growth rate. The result indicated that both factors had significant influences on the growth rate, but no interaction was detected between the two factors (Table 2).

The photosynthetic efficiency of *S. obliquus* was affected by different P concentrations with or without *Daphnia* filtrate. The maximal efficiency of PSII photochemistry (F_v_/F_m_) of *S. obliquus* was not significantly different between the control and *Daphnia* filtrate groups (F = 0.0117, *p* = 0.914). A three-way ANOVA revealed significant positive effects of time (F = 5.475, *p* = 0.002) and P concentration on F_v_/F_m_ (F = 7.115, *p* < 0.001). Consistent with the ratio of F_v_/F_m_, *Daphnia* filtrate had no significant influence (F = 0.756, *p* = 0.387) on the effective quantum yield of PSII (ΦPSII), but ΦPSII varied significantly with both time (F = 7.283, *p* < 0.001) and P concentration (F = 6.648, *p* < 0.001). In the two low P concentration treatments (0.05 and 0.2 mg L^−1^), ΦPSII decreased from day 5. ETR_max_ showed an increase on day 3. No significant variation was observed between control and *Daphnia* filtrate groups. Significant effects of time (F = 39.447, *p* < 0.001) and P concentration (F = 10.452, *p* < 0.001), as well as significant interactions between the two factors (F = 2.758, *p* = 0.001), were observed.

### Inducible defense of *Scenedesmus* under different light intensities

**Figure 4:**
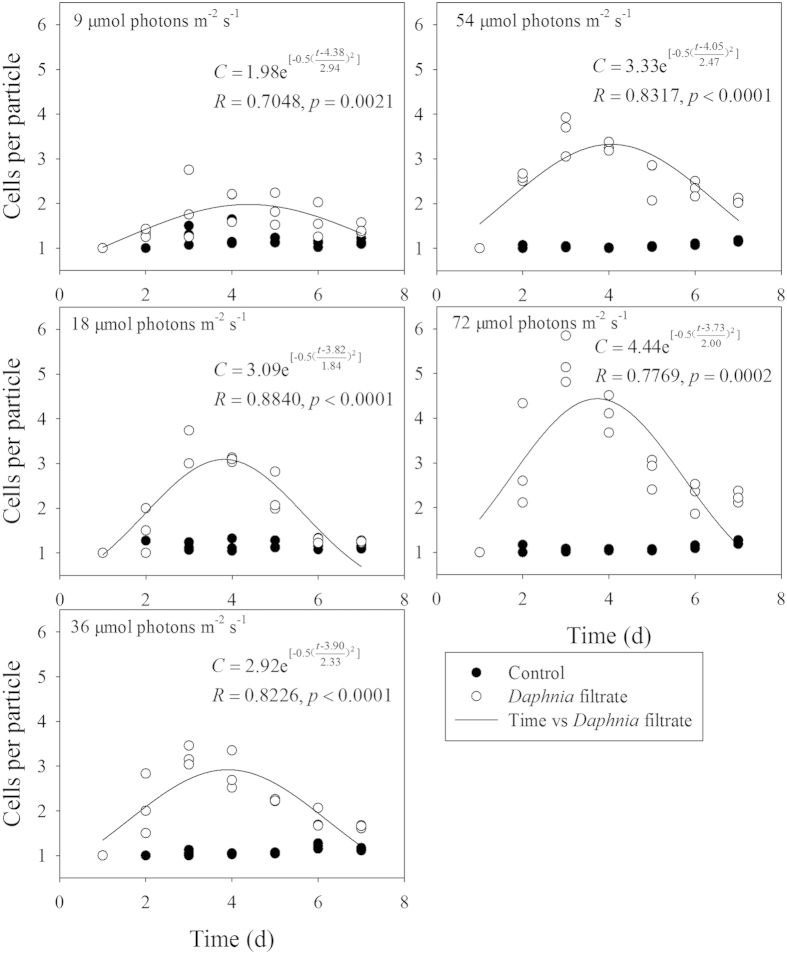
Mean number of cells per particle (*C*) of *S. obliquus* in the control or *Daphnia* filtrate groups at different light intensities. *C* changed with culture time (*t*) in *Daphnia* filtrate groups fitted by Gaussian distribution.

**Figure 5:**
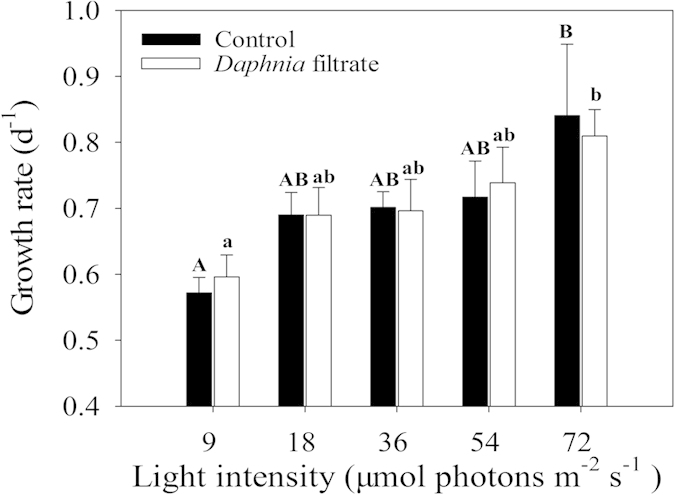
Growth rates of *S. obliquus* in the control and *Daphnia* filtrate groups at different light intensities. Vertical lines represent ±1 SE (*n* = 3). Different capital letters above solid bars denote significant (*P* < *0.05*) differences among control groups, while different lowercase letters above hollow bars denote significant (*P* < *0.05*) differences among *Daphnia* filtrate groups.

Similar to the effect of P concentration on inducible defense of *S. obliquus*, light intensity had significant influence on colony formation (Table 1). The number of cells per particle was fitted by Gaussian distribution under five light intensities. An increasing trend of the inducible defensive colony formation was observed with increased illumination (Fig. 4). Unlike the hyperbolic relationship between *C*_*max*_-observed and P concentration, *C*_*max*_-observed in response to light intensity matched the linear models (Fig. 2b). One-way ANOVA on *C*_*max*_ indicated significant light intensity effect (F = 22.928, p < 0.001). *C*_*max*_ of *S. obliquus* at 9 μmol photons m^−2 ^s^−1^ was less than half of *C*_*max*_ at 72 μmol photons m^−2 ^s^−1^. However, no significant difference of *C*_*max*_ was observed under the light intensities within the range of 18–54 μmol photons m^−2^ s^−1^. The decreased growth rate of *S. obliquus* at 9 μmol photons m^−2^ s^−1^ indicated growth inhibition by low light intensity (Fig. 5). Results of two-way ANOVA showed that different light intensities significantly affected the growth rate of *S. obliquus*, but the addition of *Daphnia* filtrate had no significant influence (Table 2). The growth rate of *S. obliquus* under the highest P concentration in the different P gradient experiment (at the light level of 45 μmol photons m^−2^ s^−1^) was lower than that in the light intensity experiment. This was possibly due to the higher initial cell density in the P gradient experiment, and the decrease in intracellular phosphorus quota because of pre-treatment of algal cells in non-phosphorus medium.

The photosynthetic efficiency of *S. obliquus* changed with light intensity with or without *Daphnia* filtrate. Three-way ANOVA indicated that the ratio of F_v_/F_m_ was influenced significantly by time (F = 4.261, *p* = 0.008) and light intensity (F = 5.017, *p* = 0.001). The ratio of F_v_/F_m_ increased with increase in light intensity. At the highest light intensity, the initial F_v_/F_m_ was high, but decreased with cultural time. Moreover, a time × light intensity interaction (F = 1.946, *p* = 0.041) was found. No significant *Daphnia* filtrate effect was detected (F = 0.702, *p* = 0.405). The three factors all affected the ΦPSII of *S. obliquus* significantly (contrast by a three-way ANOVA, time: F = 13.029, *p* < 0.001; light intensity: F = 3.927, *p* = 0.006; *Daphnia* filtrate: F = 4.609, *p* = 0.035). ΦPSII was negatively affected by the presence of *Daphnia* filtrate—it decreased significantly on day 3 at 9 μmol photons m^−2^ s^−1^, and on day 5 and 7 at 54 and 72 μmol photons m^−2^ s^−1^. Significant time × light intensity (F = 10.408, *p* < 0.001) and light × *Daphnia* filtrate (F = 4.132, *p* = 0.009) interactions were also observed. For ETR_max_, significant time (F = 13.057, *p* < 0.001) and light intensity (F = 7.042, *p* < 0.001) effects and time × light intensity interaction (F = 2.414, *p* = 0.010) were detected. The effect of *Daphnia* filtrate on ETR_max_ was not statistically significant (F = 3.123, *p* = 0.081).

## Discussion

In this study, we investigated the grazer-induced morphological defense of *S. obliquus* and the effect of resource gradient on this defense response. At all phosphorus concentrations and light intensity treatments, *Daphnia* filtrate induced the defensive colony formation of by *S. obliquus*. Consistent with previous studies^18^,^26^, the numbers of cells per particle increased under the exposure of *Daphnia* filtrate and then decreased, possibly because of the increase of cell densities and the degradation of chemical cues. Colony formation in response to *Daphnia* infochemicals exhibited a decreasing trend with decreasing environmental resource availability (Fig. 2). A strong two-way interaction between resource availability (phosphorous concentration or light intensity) and *Daphnia* filtrate indicated that the character of phenotypic plasticity of *S. obliquus* was determined by the complex interactive effects of biotic and abiotic factors, which is consistent with previous studies. Alteration of anti-predation response depending on environmental resource availability has also been observed in many other species of photoautotrophs^27^,^28^,^29^ and preys at higher trophic levels^30^,^31^.

The formation of defensive colony is highly effective for withstanding grazing pressure and preserving biomass^10^,^32^. However, limitations and costs should be expected, otherwise colony formation will be a constitutive defense^33^. To avoid excess costs of defense, many organisms adjust the intensity of inducible defenses based on predation risk and conspecific density^30^,^34^,^35^, indicating trade-offs between the benefits and costs of inducible defense. The “plant defense hypotheses”^36^ posits that decline of defensive colony formation along the resource availability gradient. In the present study, the decrease in growth rate under low resource availability condition indicates that inducible defense is accompanied by some limitations and costs.

Both light limitation and phosphate deficiency impaired the grazing-induced defense in *S. obliquus*. The negative correlations between resource availability and colony formation in *S. obliquus* may be due to resource-based trade-offs in the allocation of limiting resources. It has been already known that extracellular polysaccharides play an important role in increasing the stickiness of algal cells to form coenobia and to aggregate together. Biosynthesis of polysaccharides requires more energy and carbohydrate allocation. Therefore, increasing the production of extracellular polysaccharides will require extra investment from the intracellular pools of carbon. Limitation of external P_i_ (inorganic phosphate) will reduce the intracellular concentrations of P_i_ and ATP synthesis^37^. For PSII, the electron flow and carbon fixation rate in the algae exhibit linear correlation^38^, and the decrease of PSII electron transport rate will inhibit the biosynthesis of polysaccharides. Deficiency of external inorganic phosphate supply affects the photosynthetic apparatus of phytoplankton^39^ because a part of the light energy is allocated for nutrient uptake instead of carbon fixation^40^. With P_i_ deficiency, photosynthesis is inhibited because of decreased ribulose-1,5-bisphosphate pool size^41^, and chlorophyll content is also lower^42^, thereby causing decreased carbon assimilation area in algal cells. In the present study, P-limitation had a significant negative effect on the ratio of F_v_/F_m_ and value of ΦPSII, probably suggesting stress on PSII reaction centers under low P concentrations. ETR_max_ was also decreased under low P concentrations, suggesting that efficiency of the electron transport chain was affected. Although no significant *Daphnia* filtrate effect was observed on photosynthetic parameters, growth reduction emerged under phosphorous deficiency, specifically under the lowest P concentration, which is a relatively low level in eutrophic lakes^43^. Growth inhibition of induced algae was possibly due to increased requirement of extracellular polysaccharides to form defensive colonies under phosphate deficiency, suggesting the direct costs associated with inducible defense of *S. obliquus.*

The influence of light intensity on the defensive colony formation of *S. obliquus* was similar to that of phosphorous concentration gradient. In this study, the inducible colony formation decreased significantly at a light intensity of 9 μmol photons m^−2^ s^−1^ (Fig. 4), indicating that the ability of *S. obliquus* to form defensive colonies was inhibited under light limitation, which may be due to decreased production of carbohydrate when light intensity is low^44^. Biomass growth, carbon fixation rate, and carbohydrate productivity of phytoplankton increase with increasing light intensity below the light saturation point^45^. Under low light intensity, algal cells accumulate more lipid than carbohydrates, whereas high light intensity causes carbohydrate accumulation and cellular lipid content decreases^46^. As the key factor of photosynthesis, light has a major influence on photosynthetic carbon fixation, energy synthesis, and nutrient consumption rate^47^. The observed decrease in ΦPSII affected by *Daphnia* filtrate indicated the potential package effect of forming colonies. ETR_max_ was notably low under the lowest light intensity, which indicated strong inhibition of electron transport efficiency. Biomass production was reduced due to decrease of photosynthetic efficiency, and no efficient energy or carbohydrate source was available to maintain biomass growth for defensive colony formation. Therefore according to our study, despite the high selection pressure of herbivores, deficiency of resource availability is likely to be the limitation for *S. obliquus* in forming defensive colonies, and trade-offs between defense and development will occur. Furthermore, the reduction of photosynthetic efficiency of *S. obliquus* under low resource conditions may limit energy allocation for defense.

However, contradictory evidence on the trade-offs of resource allocation between growth, reproduction, and defense has been reported^28^,^48^,^49^,^50^,^51^,^52^,^53^. The resource availability hypothesis predicts that large investment on anti-herbivore defenses is favorable when resources are limited^48^,^49^. However, the carbon–nutrient balance hypothesis assumes resource allocation between production of defensive compounds and plant growth^50^. The variations of allocation costs associated with inducible defense may depend on the kind of deficient resources^28^,^51^, type of defense (different defensive mechanisms may have different potential sources of costs^52^), and biosynthesis of defensive compounds^53^. Considering the defensive mechanism of *S. obliquus*, poor availability of phosphorous and light will have negative effect on colony production. Colony formation in *S. obliquus* induced by FFD-6, a type of anionic surfactant, also decreases in nutrient-limited cultures^54^. Unlike *S. obliquus, S. acutus* produces colonies constitutively when phosphorous is sufficient despite *Daphnia* cues, but only produces colonies after perceiving *Daphnia* cues when phosphorus is limited^22^, indicating that the cost of forming colonies may be high and that nutrient availability does affect colony formation. Although allocation of limited resources (phosphorus and light) to growth and reproduction instead of resistance would be favored by *S. obliquus* in order to avoid physiological cost, maintenance of defensive colony under phosphorus deficiency decreased *S. obliquus* growth. Growth reduction was also detected in *Phaeocystis globosa* which formed grazer-induced colonies in low nutrient conditions, indicating the existence of costs^29^.

Despite the internal physiological costs of forming anti-grazing morphs of *S. obliquus*, previous studies also indicated the existence of other costs^23^,^25^. Maintenance of colonies reduces the ability to compete for resources and habitats with competitors^23^. Our previous study also showed that the co-existence of competitors impairs the inducible defense in *S. obliquus*^25^. Thus, these experimental studies have demonstrated the opportunity costs of defense. However, under natural conditions with complex abiotic and biotic interactions, potential ecological costs may also occur. Ecological costs are indirect consequences of induced defense and are difficult to test under laboratory conditions. Research has begun to detect the ecological costs of plants and animals (e.g., delayed flowering^55^, attracting additional natural enemies^56^, trade-offs between resistance and tolerance^57^, and other kinds of ecological costs). For phytoplankton, forming large-sized colonies is an efficient morphological defense against small herbivores. However, this defensive morph will be less effective when encountering grazers with large gape size^15^. The enhanced sinking rate of colonial *S. obliquus* will be disadvantageous for the alga to remain suspended in upper warm and euphotic waters^10^.

In conclusion, the expression of inducible defensive colony in *S. obliquus* shows dynamic responses on the availability of main environmental resources (phosphate and light). Resource insufficiency limits the expression of inducible defensive colony formation, thereby suggesting the existence of trade-offs of intercellular resource allocation between defense and growth. Our observation of growth inhibition of defensive algae under low phosphorus condition suggests that the costs may be detected under specific conditions, such as nutrient deficiency. Studying the costs accompanying the benefits of inducible defense is highly important for understanding the mechanism and evolution of inducible defense. Our work provides evidence supporting the existence of costs and trade-offs of inducible defense in *S. obliquus*.

## Methods and Materials

### Algal culture

The freshwater green alga *Scenedesmus obliquus* FACHB-416 (Chlorococcales, Chlorophyceae) can distinctly respond to infochemicals from grazers, such as cladoreans *Daphnia* spp. and rotifers. This alga is a common species used for inducible defense studies. *S. obliquus* was cultured with a modified algal growth medium BG–11 in a climate-controlled chamber at a constant temperature of 25 ± 0.5 °C with a light–dark period of 14:10 h.

### Preparation of grazer infochemicals

The chemical cues that can induce anti-grazing colony formation in *Scenedesmus* exist in the water of herbivorous zooplanktons such as *Daphnia*, which feeds on *Scenedesmus*^5^,^6^. Therefore, addition of filtered water from *Daphnia* culture was used as a common method to induce colony formation. In our experiments, the grazers *Daphnia magna* from a laboratory clone was incubated at a density of 300 individuals per liter for 24 h feeding on *S. obliquus* at a density of 10^5 ^cells ml^−1^. Then, the water was filtered through a 0.1 μm membrane filter (Millipore Corporation, USA) to eliminate bacteria and other impurities. Another suspension of *S. obliquus* without *D. magna* was filtered as control water. In preparing both control and *D. magna* water filtrates, phosphate was first excluded during culture medium preparation, and was added back at different concentrations after feeding for 24 h as outlined below.

### Experimental procedures

Five phosphorus concentrations and five light intensity levels were established to investigate the potential costs and trade-offs of forming defensive colonies. In the experiment involving different phosphorus (P) concentrations, modified BG–11 culture media with ammonium as nitrogen source were prepared at 0.05, 0.2, 0.5, 1.0, and 5.0 mg L^−1^ of P. K_2_HPO_4_ was applied as phosphorous resource and KCl was added to compensate for potassium shortage. The measured initial P concentrations in control groups were 0.049 (±0.009), 0.198 (±0.032), 0.515 (±0.029), 1.059 (±0.007), and 5.028 (±0.225) mg L^−1^, and in *Daphnia* filtrate groups were 0.050 (±0.004), 0.201 (±0.004), 0.525 (±0.013), 1.033 (±0.034), and 5.039 (±0.153) mg L^−1^. After the 7-day experiments, P concentrations decreased to 0.021 (±0.005), 0.060 (±0.011), 0.198 (±0.011), 0.719 (±0.007), 4.307 (±0.225) mg L^−1^ in control groups and 0.019 (±0.005), 0.069 (±0.006), 0.201 (±0.005), 0.723 (±0.050), 4.291(±0.125) in *Daphnia* filtrate groups. The measured initial nitrogen concentrations in control and *Daphnia* filtrate groups were 254.25 (±3.70) and 256.29 (±43.97) mg L^−1^, and it decreased to 226.17 (±34.57), 207.04 (±63.85), 227.19 (±10.06), 227.39 (±13.52), 209.48 (±16.38) mg L^−1^ in control groups and 236.55 (±7.54), 223.52 (±12.81), 221.69 (±6.54), 224.95 (±8.59), 217.62 (±24.30) mg L^−1^ in *Daphnia* filtrate groups. The total phosphorus and nitrogen concentrations were detected by the ammonium molybdate spectrophotometric method and the alkaline potassium persulfate digestion UV spectrophotometric method (China SEPA)^58^. The experimental cultures were maintained at 25 °C and illuminated at normal light level of 45μmol photons m^−2^ s^−1^ using fluorescent lights on a 14:10 light:dark cycle. Before the experiment, algal cells were starved in non-phosphate conditions for 3 days to eliminate the interference of intracellular phosphate quota. Starved algal cells were then washed with non-phosphate BG–11 medium, centrifuged at a speed of 662 g for 20 min, and placed into 250 ml Erlenmeyer flasks with different phosphorous concentrations. The initial cell density was about 7.0 × 10^4 ^cells ml^−1^.

In the experiment involving different light intensities, culture flasks were placed separately in climate-controlled chambers at five levels of light intensity, i.e., 9, 18, 36, 54, and 72 μmol photons m^−2^ s^−1^. Algal cultures were accommodated at respective light intensities for 3 days in advance^59^. The initial cell density was about 2.0 × 10^4 ^cells ml^−1^. The experimental cultures were also maintained at 25 °C on a 14:10 light:dark cycle. Both experiments had control groups and *Daphnia* filtrate-treated groups. *Daphnia* filtrate-treated groups consisted of 200 ml culture systems of *S. obliquus* at different P levels or light intensities with 10% (v/v) filtered *Daphnia* water. Control groups contained 10% filtered control water instead of *Daphnia* water. The measured initial P concentrations of control groups and *Daphnia* filtrate groups were 5.442 (±0.162) and 5.412 (±0.105) mg L^−1^. After the 7-day experiments, P concentrations decreased to 5.018 (±0.339), 4.902 (±0.357), 4.595 (±0.160), 4.691 (±0.214), 4.529 (±0.157) mg L^−1^ in control groups and 4.882 (±0.399), 4.600 (±0.076), 4.736 (±0.193), 4.761 (±0.104), 4.489 (±0.151) in *Daphnia* filtrate groups. The measured initial N concentrations in control and *Daphnia* filtrate groups were 254.66 (±25.20) and 259.95 (±35.77) mg L^−1^, and it decreased to 230.04 (±43.19), 224.14 (±13.56), 211.93 (±10.06), 229.22 (±11.99), 211.31 (±10.32) mg L^−1^ in control groups and 234.31 (±5.73), 230.24 (±9.04), 223.12 (±7.88), 218.44 (±11.14), 216.81 (±9.54) in *Daphnia* filtrate groups. All of the experimental cultures were established in 250 ml Erlenmeyer flasks. Erlenmeyer flasks were rotated artificially twice a day to keep the algal cells in suspension.

### Experimental measurements

Samples (1 ml) of every control and *Daphnia* filtrate-treated groups were taken at 9:00 a.m. every day under aseptic conditions. Lugol’s iodine solution was added at 2% as preservative after sampling. Two major quantitative traits, namely, population growth and colony size, were measured. Population growth was calculated based on cell density, which was counted using a hemocytometer (0.1 mm deep) on an Olympus microscope (Olympus 6V20WHAL; Tokyo, Japan). Eighteen 0.1 mm^3^ count areas of one sample were observed and analyzed. Growth rate was obtained as the mean slope of *ln* (*S. obliquus* cells ml^−1^) over time. The mean numbers of cells per particle were also calculated from the above counts (cell abundances and cells in different morphological particles). The number of cells per particle (*C*) versus cultural time (*t*) at all P concentrations and light intensities were fitted by using a Gaussian distribution:


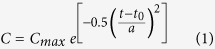


where *C*_*max*_ is the maximum number of cells per particle, *t*_*0*_ is the time to reach *C*_*max*_, and *a* is the full width at half maximum intensity of the curve. To determine the relationship between resource availability and defensive colony formation, the observed number of *C*_*max*_ was plotted against P concentration and light intensity.

Samples (2 ml) of each group were taken every day for the measurement of photosynthetic efficiency with the PHYTO-PAM phytoplankton analyzer (Heinz Walz GmbH, Effeltrich, Germany). The maximal efficiency of PSII photochemistry was determined as F_v_/F_m_, where F_v_  = (F_m_ − F_0_), and F_m_ and F_0_ are the maximal and minimal chlorophyll fluorescence yield of a dark-adapted suspension, respectively. The effective quantum yield of PSII (ΦPSII) was calculated according to the following expression: (F’_m_ − F_s_)/F’_m_, where F_s_ and F’_m_ are the stable and maximal chlorophyll fluorescence in light-acclimated algal suspensions, respectively. The electron transport rate (ETR) versus irradiance (PAR) curve was plotted for 20 different PARs within the range of 0–2000 μmol protons m^−2^s^−1^. The maximal electron transport rate (ETR_max_) was determined from a curve after fitting it to the model proposed by Platt *et al*.^60^.

### Statistical analyses

All values are presented as mean ±SE. Growth rates and numbers of cells per particle were compared by two-way ANOVA with P concentration or light intensity and *Daphnia* filtrate as the fixed factors. Three-way ANOVA was used to compare the differences and interactions among different environmental resource levels (P or light separately), *Daphnia* filtrates, and time. All data were analyzed using SigmaPlot 11.0.

## Additional Information

**How to cite this article**: Zhu, X. *et al*. Costs and trade-offs of grazer-induced defenses in *Scenedesmus* under deficient resource. *Sci. Rep.* **6**, 22594; doi: 10.1038/srep22594 (2016).

## Figures and Tables

**Table 1:** Summary of two-way ANOVA for *Daphnia* filtrate and phosphorous concentration or light intensity on colony formation of *Scendesmus obliquus.*

Factors	Source of Variation	DF	SS	MS	F	P
Phosphorus concentration	Time	6	27.156	4.526	177.139	<0.001
	Phosphorus concentration	4	1.24	0.31	12.137	<0.001
	*Daphnia* filtrate	1	32.276	32.276	1263.222	<0.001
	Time × P conc.	24	2.61	0.109	4.256	<0.001
	Time × *Daphnia* filtrate	6	9.518	1.586	62.089	<0.001
	P conc. × *Daphnia* filtrate	4	1.333	0.333	13.039	<0.001
	Time × P conc. × *Daphnia* filtrate	24	1.4	0.0583	2.283	0.002
Light intensity	Time	6	32.775	5.463	74.927	<0.001
	Light intensity	4	10.155	2.539	34.823	<0.001
	*Daphnia* filtrate	1	66.681	66.681	914.63	<0.001
	Time × Light intensity	24	5.816	0.242	3.324	<0.001
	Time × *Daphnia* filtrate	6	29.628	4.938	67.733	<0.001
	Light intensity × *Daphnia* filtrate	4	12.867	3.217	44.122	<0.001
	Time × Light intensity × *Daphnia* filtrate	24	7.934	0.331	4.534	<0.001

**Table 2:** Summary of two-way ANOVA for *Daphnia* filtrate and phosphorous concentration or light intensity on growth rate of *S. obliquus.*

Factors	Source of Variation	DF	SS	MS	F	P
Phosphorus concentration	Phosphorus concentration	4	0.131	0.0328	26.687	<0.001
	*Daphnia* filtrate	1	0.00673	0.00673	5.483	0.030
	P conc. × *Daphnia* filtrate	4	0.00228	0.000569	0.463	0.762
Light intensity	Light intensity	4	0.179	0.0447	5.585	0.003
	*Daphnia* filtrate	1	2.73E-05	2.73E-05	0.00341	0.954
	Light intensity × *Daphnia* filtrate	4	0.00304	0.00076	0.095	0.983
